# Application of perturbation gene expression profiles in drug discovery—From mechanism of action to quantitative modelling

**DOI:** 10.3389/fsysb.2023.1126044

**Published:** 2023-02-09

**Authors:** Bence Szalai, Dániel V. Veres

**Affiliations:** ^1^ Turbine Ltd., Budapest, Hungary; ^2^ Department of Molecular Biology, Semmelweis University, Budapest, Hungary

**Keywords:** gene expression, perturbation, mechanism of action, drug repurposing, pathway activity, modelling

## Abstract

High dimensional characterization of drug targets, compound effects and disease phenotypes are crucial for increased efficiency of drug discovery. High-throughput gene expression measurements are one of the most frequently used data acquisition methods for such a systems level analysis of biological phenotypes. RNA sequencing allows genome wide quantification of transcript abundances, recently even on the level of single cells. However, the correct, mechanistic interpretation of transcriptomic measurements is complicated by the fact that gene expression changes can be both the cause and the consequence of altered phenotype. Perturbation gene expression profiles, where gene expression is measured after a genetic or chemical perturbation, can help to overcome these problems by directly connecting the causal perturbations to their gene expression consequences. In this Review, we discuss the main large scale perturbation gene expression profile datasets, and their application in the drug discovery process, covering mechanisms of action identification, drug repurposing, pathway activity analysis and quantitative modelling.

## Introduction

Identification of the systems level alterations in diseases and their relationships to drug effect and efficacy are crucial to better understand drug-disease relationships and develop new therapeutics (X. Yang et al., 2020). The most frequently used high-throughput, genome wide (“omics”) methods for such a systems level characterisation are still transcriptomic measurements such as microarray and RNAseq (McGettigan 2013; Manzoni et al., 2018). Despite the relatively affordable acquisition and well established analysis methods, the correct interpretation of gene expression measurements are complicated by several factors. Classical analysis methods return lengthy lists of differentially expressed genes (e.g., healthy vs. control sample), however differential expression does not necessarily mean altered activity on protein level (Nusinow et al., 2020; Piran et al., 2020), and also differentially expressed genes are frequently not the cause, but the consequence of the investigated phenotype. While different prior-knowledge based bioinformatics methods like pathway analysis techniques (Nguyen et al., 2019) can help in the interpretation, identifying the causal alterations are still difficult.

**FIGURE 1:**
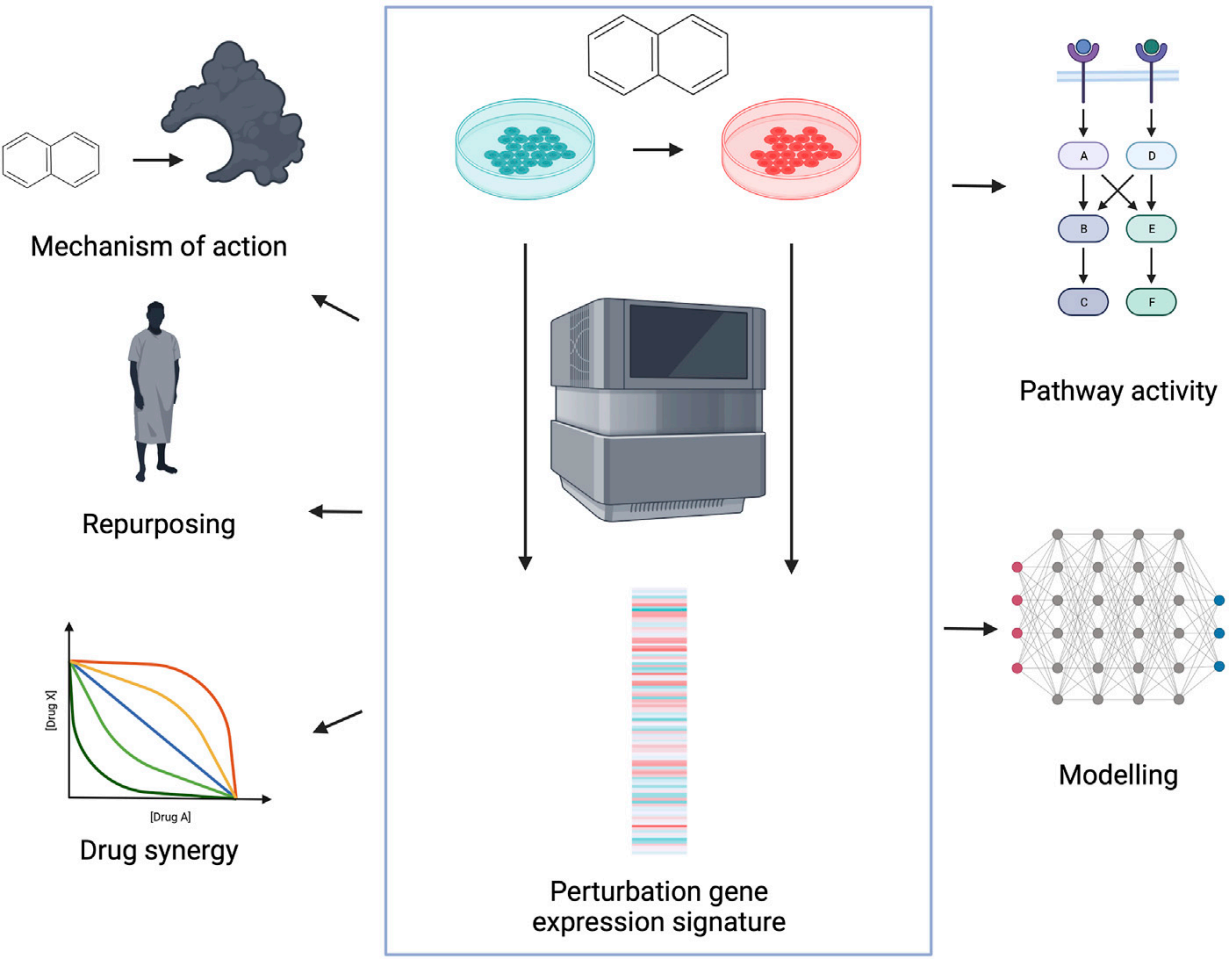
Application of perturbation gene expression profiles in drug discovery. Perturbation gene expression signatures are defined as differential expression (DE) signatures between perturbed and control samples (middle). Signatures can be used to identify compound mechanisms of action, to repurpose existing drugs for new indications, to identify synergistic combinations and for mechanistic understanding and following modelling of the drug induced perturbation phenotypes. Created with BioRender.com.

Perturbation gene expression signatures are defined as the gene expression difference between a perturbed and control condition, calculated by differential expression analysis (Love et al., 2014; Ritchie et al., 2015). In case of perturbation signatures, we can directly connect the cause (perturbation) and the downstream effect (gene expression signature), which can help to understand cellular mechanisms (Lamb et al., 2006). Perturbation gene expression profiles can be generated on gene level (by knocking-out/down or overexpressing the gene of interest) and also on compound level. Analysis of drug induced perturbation signatures can help in several steps of the drug discovery process (Figure 1). Comparison of drug and gene related gene expression profiles can highlight drug mechanisms of action, and identify potential off-targets. Comparison of disease signatures (differential expression of disease and corresponding healthy tissue) and drug signatures is frequently used to identify new indications for existing drugs. Analysing signatures of drug pairs can identify synergistic and antagonistic drug combinations. Finally, perturbation signatures can help to develop methods to gain mechanistic understanding of cellular processes from omics data.

In this Review, we discuss the most frequently used perturbation transcriptomics measurement methods and the corresponding large scale dataset, and the above mentioned main applications, with a focus on cancer drug discovery.

## Perturbation gene expression measurement methods and datasets

While large amounts of low-scale perturbation gene expression datasets are available in various public repositories [like GEO (Barrett et al., 2013) and ArrayExpress (Kolesnikov et al., 2015)], their usability is hindered by the complicated searchability, lack of uniform metadata format and possible batch effects between studies. To overcome these problems, several authors created secondary databases, where gene expression profiles from these public repositories were collected, uniformly preprocessed and even metadata is standardised (Lachmann et al., 2018). Crowdsourcing methods and natural language processing (NLP) techniques can help to speed up the lengthy manual metadata curation in these cases (Z. Wang et al., 2016). Generally, a large proportion of these collected datasets are focusing on transcriptomics changes in cancer cell lines, but there are also dedicated collections of gene expression signatures from non-cancerous tissues (Zheng et al., 2022).

Connectivity Map (Lamb et al., 2006) was the first large-scale attempt to create a compendium of gene expression signatures. Connectivity Map used originally 164 small molecules as perturbations in 4 cell lines, and gene expression was measured with Affymetrix microarrays. While this dataset was used in several studies investigating drug mechanism of action and repurposing, Connectivity Map lacked both genetic perturbations and diversity of cell lines. High cost of microarray and bulk RNA sequencing made perturbation gene expression profile generation unscalable, thus the authors of the original Connectivity Map created the “next-generation” Connectivity Map by measuring a reduced transcriptome, using the L1000 technology (Subramanian et al., 2017). Hybridisation based L1000 assay measures only the expression of 978 “landmark” genes, and the rest of the transcriptome is computationally inferred. L1000 technology allowed the generation of more than 1,000,000 perturbation gene expression profiles until 2017, and a new >3,000,000 profile dataset is also available (at https://clue.io/). Importantly, the new Connectivity Map contains more cell lines and a more diverse collection of perturbations, including genetic (shRNA, CRISPR, and overexpression), chemical (small molecules) and physiological (ligand) perturbations.

While RNA sequencing was considered too expensive to generate large scale perturbation gene expression profiles, recent technological advancements, especially single cell sequencing methods, brought about a change in this. By using barcoding and pooling strategies, bulk sequencing methods like PLATE-Seq (Bush et al., 2017) and DRUG-Seq (Ye et al., 2018) reached comparable costs to the L1000 assay and allowed the production of large scale drug perturbation screens like PANACEA (Douglass et al., 2022). Recent advancement of single cell RNA sequencing (scRNA-seq) methodologies further increased the throughput of perturbation gene expression profiling. One of the pioneer methods of the field, Perturb-Seq combines CRISPR (Dixit et al., 2016) or CRISPRi (Adamson et al., 2016) and sc-RNAseq with the help of expressed guide barcodes. Perturb-Seq allows genome-wide (Replogle et al., 2022) genetic perturbations, but only in one cell line in each experiment. Other methods, like MIX-Seq (McFarland et al., 2020), use a smaller number of chemical perturbations (anti-cancer drugs), but measure gene expression profiles in a large number of cell lines, with the help of SNP-based computational demultiplexing. Importantly, scRNA-seq based methods can also identify heterogeneity of perturbation response. As the number of scRNA-seq perturbation screens increases, it is important to categorise and harmonise these datasets, as done in (Peidli et al., 2022).

Currently a large number of ultra-high throughput methods are available for new hybridization (L1000) and bulk/single cell RNA-seq based perturbation gene expression screens. Also, the previously described (Table 1), public datasets give rich sources for *in silico* analysis of existing results. However, it is important to highlight that all of these different methods and datasets have their own intrinsic biases, highlighting the importance of harmonisation and comparison of results from different sources. First important factor to consider is the type of the used assay: L1000 measures only a reduced part of the transcriptome, which can only explain partial variance (∼90%) of total transcriptomics difference (Subramanian et al., 2017). While the rest of the transcriptome is inferred, the computationally inferred transcriptomic changes are less reliable, especially in case of very specific perturbations (e.g., shRNA induced gene expression decrease of the target gene is generally only detected in case of landmark genes, and not in case of inferred genes). Single cell sequencing also leads to lower number of detected genes than classical bulk sequencing, however pseudo-bulking methods can help to overcome these problems (McFarland et al., 2020). While lower coverage in case of L1000 and scRNAseq based assays can be problematic to identify specific differentially expressed genes, multigene signature based techniques (discussed in the following sections) are less sensitive for the lower number of detected genes (Holland et al., 2020). It is also important to consider perturbation type related differences in case of interpretation. While CRISPR has the highest specificity, shRNA perturbations can lead to partial inhibition, which resembles drug effect better in some cases (Michael Krill-Burger et al., 2022). In case of drug perturbations, increasing the drug concentration can lead to higher proportion of off-target effects, and (especially in case of oncology drugs) increased toxicity, which can mask the compound specific transcriptional effect (Szalai et al., 2019).

**TABLE 1:** Public datasets and databases for perturbation gene expression profiles.

Dataset	Perturbation type	Short description	Database URL	Reference
CREEDS	chemical and genetic	crowdsourced collection of perturbation signatures from GEO	https://maayanlab.cloud/CREEDS/	Wang et al. (2016)
ChemPert	chemical	collection of non-cancer perturbation signatures	https://chempert.uni.lu/	Zheng et al. (2022)
Connectivity Map (LINCS)	chemical and genetic	L1000 assay based, >3,000,000 signatures	https://clue.io/	Subramanian et al. (2017)
PANACEA	chemical	anti-cancer drug perturbation signatures in multiple cell lines, RNAseq		Douglass et al. (2022)
Perturb-Seq (genome wide)	genetic	genome-wide CRISPRi in 1 cell line, scRNA-seq	https://gwps.wi.mit.edu/	Replogle et al. (2022)
Mix-Seq	chemical	anticancer drug perturbations in multiple cell lines, scRNA-seq		McFarland et al. (2020)

## Mechanism of action inference

As potential off-targets can influence both adverse effects and clinical efficacy (Lin et al., 2019), characterisation of drug target profiles is a crucial step of drug development. Also, identifying new targets of existing drugs can facilitate drug repurposing in new indications (see also next section). Classical methods characterise drug targets by the binding strength of drugs to individual target proteins. While these methods can effectively characterise the binding characteristics to the main targets (or shortlisted off-target candidates), they are not feasible on genome/proteome scale to identify off-targets. In contrast, gene expression changes induced by drug perturbations can help to define target profiles potentially on the genome scale (Trapotsi et al., 2022).

**FIGURE 2:**
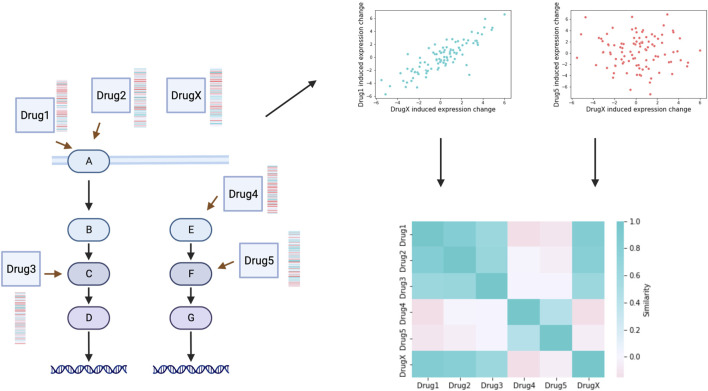
Mechanism of action inference. Similarity (correlation or enrichment based) can be calculated between the perturbation gene expression profiles of drugs with known MoA (Drug1-Drug5) and a compound with unidentified MoA (DrugX). Similarity matrix indicates mechanism of action relationships of drugs, and can be used to identify MoA of the unknown compound. Created with BioRender.com.

The basic principle of gene expression profile based mechanism of action (MoA) identification is based on the fact that compounds with shared mechanisms of action lead to similar changes of cellular signalling mechanisms, thus leading to similar gene expression changes (Figure 2). The perturbation gene expression profile of a drug with unknown mechanism of action can be used to query large scale datasets (see previous section) and identify potential MoA based on similarities (Musa et al., 2018). Frequently used similarity metrics are generally correlation (Szalai et al., 2019) or enrichment (Subramanian et al., 2017) based. Drug signature based MoA identifications has successfully used to identify Rho-kinase inhibitor *Fasudil* as autophagy inducer (Iorio et al., 2010), PKC inhibitor *Enzastaurin* as GSK3 inhibitor (Subramanian et al., 2017) and the role of JAK2 in the MoA of *Mitomycin C* (Woo et al., 2015). In a recent large scale, crowdsourced benchmarking study (Douglass et al., 2022), participants used perturbation gene expression signatures of cancer cell lines treated with 32 kinase inhibitors to predict the targets of these (for the participants unknown) drugs. Best performing methods were able to predict experimentally verified targets with ROC AUC >0.7, also confirming the applicability of gene expression signatures for genome wide inference of drug (off-) targets. With this study (Douglass et al., 2022), also created a benchmark dataset for further computational studies.

Drug signature based MoA identification has similar performance as the gold standard of the field, chemical similarity based methods (Baillif et al., 2020). However, while chemical similarity based target/MoA prediction can be performed on any compound of a (even virtual) library, expression profile similarity based predictions require prior measurement of drug induced gene expression signature. On the other hand, expression signatures can help to identify more “unexpected” off-targets and mechanism of actions. This is especially true, if the similarity is calculated between the signatures of chemical and genetic perturbations, where MoA is identified not through “guilt-by-association” (similarity to drugs with known MoA), but based on the direct similarity of a drug’s and its target’s genetic perturbation profile. Recently several methods were developed to infer compound MoA based on CRISPR and shRNA induced signatures (Pabon et al., 2019; Jang et al., 2021; Zhong et al., 2022). On the other side, time scale and efficacy of perturbation can be substantially different between chemical and genetic perturbations (e.g., in Connectivity Map (LINCS) dataset gene expression is measured 24 h after drug, but 96 h after genetic perturbation), which can make cross perturbation modality comparisons more complicated. Importantly, perturbation signatures can be generated for each investigated cell line, but “consensus” (i.e., general, cell line independent) signatures of drugs can be also created. While using consensus signatures can simplify analysis pipelines, they can mask cell line specific effects (Innes and Bader 2021), and as recent analysis suggest, some methods for consensus signature calculation can lead to artificial similarities of unrelated signatures (Smith and Haibe-Kains 2022). Also, it is important to highlight that similarity between gene expression profiles does not necessarily imply shared MoA, especially in case of anti-cancer drugs. Anti-cancer drugs lead to decreased cell viability, which is represented in their perturbation gene expression signatures (Szalai et al., 2019; Jones et al., 2020; McFarland et al., 2020). Thus two cytotoxic drugs can have similar gene expression signature, despite having distinct mechanisms of action, which effect can be removed by appropriate statistical models (Szalai et al., 2019; McFarland et al., 2020). On the other side, drug and genetic perturbation induced cell viability changes can be also used to identify the target profile of anti-cancer compounds: correlating drug sensitivity (Iorio et al., 2016; Corsello et al., 2020) and gene essentiality (Tsherniak et al., 2017; Behan et al., 2019) on large panels of cancer cell lines (Ghandi et al., 2019; van der Meer et al., 2019) can help to identify on- and off-targets based on sensitivity profile similarity (W. Wang et al., 2022; Gonçalves et al., 2020). Gene expression signature and sensitivity profile based methods can complement each other in computational MoA inference.

## Drug repurposing—Signature reversion

Drug repurposing, the process of finding new indications for existing, approved drugs, gains more and more relevance with the increasing costs of *de novo* drug development (Pushpakom et al., 2019). Importantly, already approved drugs have a lower chance to fail due toxicity in new indications. Drug repurposing is especially important in case of rare diseases, where small market size makes *de novo* drug discovery even more complicated.

Drug induced perturbation gene expression profiles are frequently used for computational drug repurposing (Sirota et al., 2011). The main hypothesis behind signature reversal based methods is, that if a drug induced gene expression signature is anti-similar to a disease related gene expression signature, then the drug can potentially reverse the disease specific gene expression changes, thus the disease phenotype. In these studies, the similarity of disease signatures and drug induced signatures is calculated, and drugs showing negative similarity are prioritised for further experimental validation. Signature reversal hypothesis - despite its relative simplicity—led to identification of repurposable drug candidates from cancer (B. Chen et al., 2017; Stathias et al., 2018) through inflammatory (Malcomson et al., 2016) to metabolic diseases (Kunkel et al., 2011).

While signature reversion is frequently used to identify anti-cancer compounds (B. Chen et al., 2017), recently the confounding role of cell proliferation in these studies have been revealed. While cancer signature (differential expression signature between cancer samples and corresponding healthy cells/tissues) contains a strong cell proliferation related component, anti-cancer drugs generally inhibit cell proliferation, and their gene expression signature contains a strong anti-proliferative (cell death related) component (Szalai et al., 2019). This suggests that the anti-similarity of cancer and anti-proliferative drug signatures is trivial, and the drugs identified by signature reversal methods are not necessarily effective in the investigated cancer type, just general toxic compounds. A recent publication (Koudijs et al., 2022) showed that removing the confounding effect of proliferation/anti-proliferation related gene expression changes significantly decreased the predictive performance of signature reversal methods.

Another disease indication, where the signature reversal hypothesis has to be used with caution, is infectious diseases. During the COVID-19 pandemic, signature reversal methods have been frequently used to identify potential antiviral drugs against SARS-CoV-2. Several of these studies found that drugs having a similar (and not anti-similar, which would be assumed based on the signature reversal hypothesis) gene expression signature to SARS-CoV-2 infection induced transcriptomics signature are effective *in vitro* antivirals (F. Chen et al., 2021; Barsi et al., 2022). As in case of viral infection diseases, infected host cells activate adaptive, antiviral pathways (like NFkB and JAK-STAT), supporting and not reversing these activities indeed can have beneficial, antiviral effects. Nevertheless, other studies found that drugs showing anti-similarity to influenza infection signature are effective antivirals (Pizzorno et al., 2019), thus the general usefulness of signature reversal methods in infection diseases needs further evaluation.

Most current signature reversal methods use bulk disease transcriptomics data for drug prioritisation. However, in a bulk tissue sample, several different cell types exist. While the gene expression changes of some of these cells can have a causal role in disease development (thus are candidates for signature reversing drugs), other cell types’ gene expression profile can change as a consequence of disease process. Using single cell RNA-seq to identify cell type specific disease signatures and repurposable drugs can further increase the applicability of signature reversal methods (Liu et al., 2022).

## Identifying synergistic drug combinations

Using drug combinations can help to use lower drug doses, thus can decrease the frequency of adverse effects, and can help to overcome drug resistance mechanisms, especially in case of anti-cancer compounds. Drug combinations are classified as synergistic, additive or antagonistic, based on the difference between observed and expected drug effects, where the expected drug effect is calculated using some synergy model like Bliss independence of Loewe additivity models (Yadav et al., 2015). To experimentally measure synergy, multiple dose—Response curve measurements are required, thus large-scale experimental testing is generally not feasible due to the combinatorial increase of the number of possible combinations. Computational methods are frequently used to infer drug synergy for new combinations in new biological samples.

Generally, machine learning models (Menden et al., 2019) use features of drugs, and features of the cell line to predict synergistic effects of the drugs. The most frequently used drug features are chemical fingerprints or other representation of the drugs chemical structure (Preuer et al., 2018). However, drug induced gene expression signatures contain more context specific biological information regarding drug effect than chemical structure, thus their application for drug synergy prediction can be beneficial. Using drug signatures has been used to predict synergistic effects of anti-cancer drugs (Bansal et al., 2014), and a recent benchmarking study showed that machine learning models using expression based features significantly outperform standard, chemical feature based methods (El Khili et al., 2022). Interestingly, several of these studies suggest that similarity between drug signatures is a strong predictor of synergistic drug effect (Diaz et al., 2020), suggesting drugs targeting the same pathway, but at different targets are generally more synergistic. Other studies found that strong compound similarities, but also dissimilarities are correlating with synergistic drug effect (M. Yang et al., 2020), suggesting that more detailed computational studies and experimental datasets are needed to fully understand mechanisms behind drug synergy. Importantly, measuring combination induced transcriptomics changes (Mathur et al., 2022) can help to resolve these ambiguities.

## Interpretation—Pathway activity

While mechanism of action, drug repurposing, and even synergy prediction is possible by similarity based/black-box machine learning methods, biological, mechanistic interpretation of drug induced gene expression changes can also help to better understand drug effect and identify potential biomarkers. Standard methods for such a biological interpretation are gene set enrichment and pathway analysis techniques (Nguyen et al., 2019).

**FIGURE 3:**
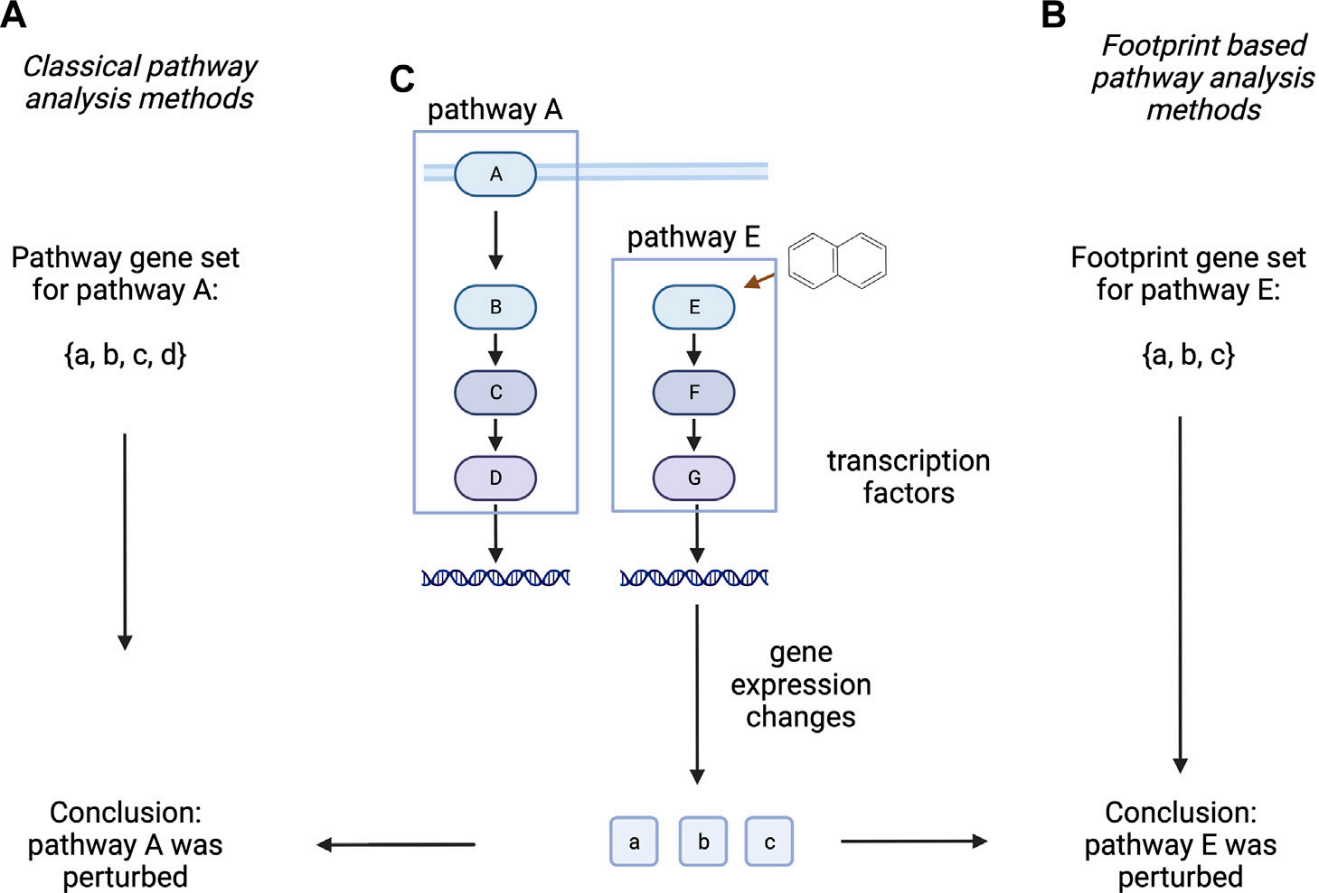
Classical and footprint based pathway analysis methods. Classical pathway analysis methods **(A)** use the gene set created from pathway member genes. Footprint based pathway analysis methods **(B)** use gene sets of pathway regulated genes. In a hypothetical experiment **(C)**, where protein E is perturbed with a drug, altered expression of a, b, c genes is measured. Classical methods infer the altered activity of Pathway A (composed of A, B, C, D proteins), while footprint based methods correctly identify Pathway E as the target of the perturbation. Proteins are labelled with uppercase while corresponding mRNA with lowercase letters. Created with BioRender.com.

Classical pathway analysis techniques calculate some kind of enrichment of differentially expressed genes, using pathway member genes as gene sets (Subramanian et al., 2005; Liberzon et al., 2015). Importantly, these methods (indirectly) assume some clear connection between gene expression, protein abundance and protein activity, however these assumptions are not necessarily correct (Szalai and Saez-Rodriguez 2020). Other methods are using the expression of pathway regulated (“footprint”) genes to infer which pathway activity changes had led to the observed gene expression pattern (A. Dugourd and Saez-Rodriguez 2019). These later methods have been shown to better represent biological phenotype in several benchmarks than classical, pathway membership based pathway analysis techniques (Cantini et al., 2018; Holland et al., 2019; Szalai and Saez-Rodriguez 2020; Douglass et al., 2022). One can argue that “footprint” based methods are better suited to identify pathway activity changes responsible for the gene expression changes of a sample, while classical, membership based methods try to infer the possible consequence of gene expression changes on pathway activity. While the latter can identify important information (like negative feedback mechanisms), it is generally mode speculative than results of “footprint” based methods (Figure 3).

Of course, to apply “footprint” based pathway analysis methods, prior knowledge regarding the pathway regulated genes is required. This information can be collected *via* literature mining and gene regulatory network inference (Alvarez et al., 2016; Garcia-Alonso et al., 2019; Keenan et al., 2019), but perturbation expression profile datasets are also excellent sources for this information. Methods like PROGENy (Schubert et al., 2018) or SPEED (Parikh et al., 2010; Rydenfelt et al., 2020) collected a large set of gene expression profiles related to the investigated pathways, and used different statistical models to identify the pathway responsible “footprint” genes (Table 2). Footprint concept has recently extended to infer ligand/receptor associations and activities (Jiang et al., 2021). Interestingly, benchmarking studies suggest (Badia-i-Mompel et al., 2022) that quality of the used footprint gene set has a higher influence on the performance, than the used statistical method. In case of transcription factor activity inference methods, the ones using multiple sources of regulatory interactions (like ChIP-Seq data, co-expression, literature curated data and promoter binding motifs) perform better (Garcia-Alonso et al., 2019; Keenan et al., 2019) than the ones relying on single sources of information. The performance of perturbation profile based pathway activity inference methods is also strongly dependent on the quality and amount of the collected perturbation experiments (Schubert et al., 2018). Perturbation expression profiles are also needed for the correct benchmarking of pathway analysis techniques, as for assessing a newly developed method, ground-truth data is required. Importantly, while for the development and benchmarking of footprint based pathway activity inference tools perturbation gene expression data is suitable, these methods can be effectively used also on baseline (e.g.,: disease samples) expression data (Schubert et al., 2018).

**TABLE 2:** Computational tools for footprint based pathway activity inference.

Tool	Inferred activity	Short description	Tool URL	Reference
ChEA3	Transcription factor	Co-expression and ChIP-seq based regulatory interactions for TFs	https://maayanlab.cloud/chea3/	Keenan et al. (2019)
CytoSig	Cytokine	Signatures collected for 43 cytokines form perturbation data	https://cytosig.ccr.cancer.gov/	Jiang et al. (2021)
DoRothEA	Transcription factor	Co-expression, motif, ChIP-seq and literature based regulatory interactions for TFs	https://bioconductor.org/packages/release/data/experiment/html/dorothea.html	Garcia-Alonso et al. (2019)
Viper	Protein	Co-expression based regulatory interactions	https://www.bioconductor.org/packages/release/bioc/html/viper.html	Alvarez et al. (2016)
PROGENy	Pathway	Signatures collected for 14 cancer related pathways from perturbation data	https://bioconductor.org/packages/release/bioc/html/progeny.html	Schubert et al. (2018); Holland et al. (2019)
SPEED2	Pathway	Signature collected for 16 pathways form perturbation data	https://speed2.sys-bio.net/	Rydenfelt et al. (2020)

## Modelling cellular phenotype

One of the main goals of systems biology studies is to develop mechanistically understandable and simulatable models of cellular processes (Froehlich et al., 2017). Importantly, virtual perturbation in these models can help to identify biomarkers and synergistic drug combinations (Eduati et al., 2017). These models generally use some prior-knowledge biological (signalling) network (Türei et al., 2021) to connect proteins (nodes) and use data-driven methods to parameterise (fit) the network parameters (edge weights) to the biological context (Garrido-Rodriguez et al., 2022). To fit the network parameters, a wide range of computational tools are used, like graph algorithms (Browaeys et al., 2019), integer linear programming (Liu et al., 2019) or neural network architectures (Yuan et al., 2021; Nilsson et al., 2022). Perturbation data is especially suitable for contextualisation of these models (Korkut et al., 2015), as in case of perturbation experiments the response of the same cellular system is measured, which lowers the amount of possible parameters of the model.

While proteomics data has been used most frequently for simulation of signalling networks (Yuan et al., 2021), recently gene expression data has been also effectively applied in this context (Table 3). Importantly, while (phospho) proteomics data can be used directly to approximate activity of signalling components, transcriptomics data is less suitable for modelling signal flow. However, either by using gene expression data in gene regulatory context (Browaeys et al., 2019; Liu et al., 2019; Fortelny and Bock 2020; Babur et al., 2021) or inferring protein activities from gene expression (Nilsson et al., 2022) can help to build effective mechanistic models for cell signalling using transcriptomics data. While perturbation data based mechanistic models are obviously hard to create for patient data, baseline data could also be used to transfer and contextualise these models to *in vivo* settings (Saez-Rodriguez and Blüthgen 2020). Currently, the main application of these modelling frameworks is hypothesis generation and more general and unbiased benchmarks are needed to compare them and measure their general predictive performance.

**TABLE 3:** Gene expression based mechanistic modells.

Tool	Short description	Tool URL	Reference
CARNIVAL	Identifies signalling pathway activity changes by connecting perturbations to inferred transcription factor activities using integer linear programming.	https://github.com/saezlab/CARNIVAL	Liu et al. (2019)
NicheNet	Predicts protein (ligand-receptor) activity by connecting receptors to gene expression through signalling and gene regulatory network. Strength of ligand—gene expression interactions are calculated *via* Personalised PageRank.	https://github.com/saeyslab/nichenetr	Browaeys et al. (2019)
KPNN	Predicts protein activity from gene expression using a neural network architect resembling gene regulatory and signalling network.	https://github.com/epigen/KPNN	Fortelny and Bock. (2020)
CausalPath	Identifies causal priors (causal graph motifs) from Pathway Commons database Cerami et al. (2011), and matches them with correlated changes from the analysed data. Uses also (phospho)proteomics data	https://github.com/PathwayAndDataAnalysis/causalpath	Babur et al. (2021)
LEMBAS	Predicts transcription factor activity from ligand stimulation using a recurrent neural network resembling signalling network.	https://github.com/Lauffenburger-Lab/LEMBAS	Nilsson et al. (2022)

## Conclusion

As collected above, perturbation gene expression profiles can give rich input data for drug discovery and development. Drug induced expression changes can help to identify the on- and off-targets of newly developed compounds, comparison to disease signatures can reveal new disease indications, and suitable analysis of gene expression signatures can give mechanistic insight regarding drug action.

**FIGURE 4:**
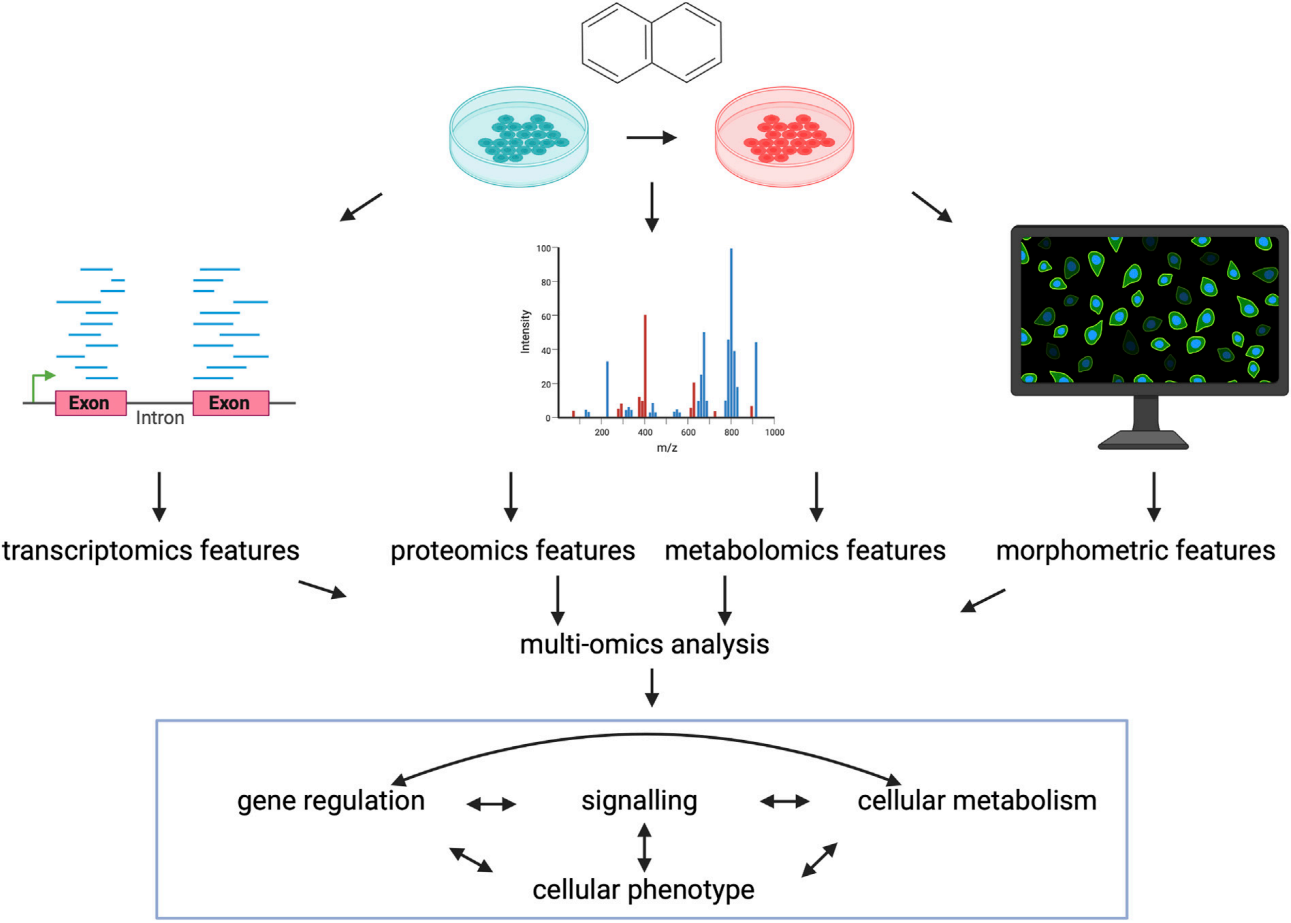
Perturbation multi-omics. Characterising the same perturbations with data from different omics-modalites can give better description of cellular phenotype and can better describe the individual layers of cellular regulation and the connections between them. Created with BioRender.com.

Additionally, recently more and more perturbation data from other omics modalities is available, suggesting the importance of perturbation multi-omics in the future. High throughput, high content imaging and featurization of images allows to derive morphological signatures of perturbed cell states, which can be used to identify compound mechanisms of action similarly to gene expression profiles (Haghighi et al., 2022). Also, morphological profiles are generally interpretable for cell biologists, and can be directly connected to perturbation induced cell states. Proteomics and phospho-proteomics are more closely connected to the activity of cellular process than gene expression, so perturbation proteomics datasets can be used to infer compound effects of signalling and compound similarities (Zhao et al., 2020; Gabor et al., 2021). Recently also drug induced metabolomics changes were used to describe cellular phenotype and synergistic drug effect (Lu et al., 2022). Figure 4.

Most importantly, different omics layers can measure different, not interchangeable variance of cellular phenotype (Gross et al., 2022), thus using integrative data analysis (Argelaguet et al., 2020) and modelling methods (Dugourd et al., 2021) of different, but harmonised omics modalities can lead to better understating of drugs’ effect on cellular processes in the future.

In summary, perturbation gene expression measurements create valuable insight to analyse durg effect in target cells, and the computational tools developed with perturbation data can also be generalised to baseline, including patient data.
